# Regional regulation of transcription in the chicken genome

**DOI:** 10.1186/1471-2164-11-28

**Published:** 2010-01-14

**Authors:** Haisheng Nie, Richard PMA Crooijmans, John WM Bastiaansen, Hendrik-Jan Megens, Martien AM Groenen

**Affiliations:** 1 Animal Breeding and Genomics Centre, Wageningen University, Marijkeweg 40, 6709 PG, Wageningen, the Netherlands

## Abstract

**Background:**

Over the past years, the relationship between gene transcription and chromosomal location has been studied in a number of different vertebrate genomes. Regional differences in gene expression have been found in several different species. The chicken genome, as the closest sequenced genome relative to mammals, is an important resource for investigating regional effects on transcription in birds and studying the regional dynamics of chromosome evolution by comparative analysis.

**Results:**

We used gene expression data to survey eight chicken tissues and create transcriptome maps for all chicken chromosomes. The results reveal the presence of two distinct types of chromosomal regions characterized by clusters of highly or lowly expressed genes. Furthermore, these regions correlate highly with a number of genome characteristics. Regions with clusters of highly expressed genes have higher gene densities, shorter genes, shorter average intron and higher GC content compared to regions with clusters of lowly expressed genes. A comparative analysis between the chicken and human transcriptome maps constructed using similar panels of tissues suggests that the regions with clusters of highly expressed genes are relatively conserved between the two genomes.

**Conclusions:**

Our results revealed the presence of a higher order organization of the chicken genome that affects gene expression, confirming similar observations in other species. These results will aid in the further understanding of the regional dynamics of chromosome evolution.

The microarray data used in this analysis have been submitted to NCBI GEO database under accession number GSE17108. The reviewer access link is: http://www.ncbi.nlm.nih.gov/geo/query/acc.cgi?token=tjwjpscyceqawjk&acc=GSE17108

## Background

Gene expression in eukaryotes is regulated on two different levels, i.e. individual gene level and regional level in the genome. The best studied, and generally considered the major level of regulation, is the regulation at the level of individual genes. Although a number of well studied exceptions have identified a number of tightly co-regulated gene clusters, such as the globin, MHC and the Hox gene gene clusters [1-4], the common model for eukaryotic gene transcription involves the binding of several transcription factors (TFs) to promoter regions and enhancers, resulting in activation of the individual genes. It has become increasingly evident that in addition to gene regulation by TF binding to regulatory sequences, eukaryotic gene expression is also regulated at a higher level, and several studies have demonstrated the dependency of gene expression on the location of the gene within the genome [5-7].

Over the past years, the relationship between gene transcription and chromosomal location has been studied in a number of different vertebrate genomes. Analysis of the human transcriptome map based on SAGE (serial analysis of gene expression) data from 12 human tissues [8] revealed the clustering of highly expressed genes within specific chromosomal regions; these regions were termed "RIDGEs", or "Regions of IncreasedGene Expression". Genomic regions containing genes expressed at much lower levels were termed anti-RIDGEs, and these regions exhibit characteristics opposite those of RIDGEs [8,9]. A similar region-wide regulation of gene expression was later reported in the *Drosophila *genome [10,11]. RIDGEs were also found in the mouse genome [12] and are reported to be relatively conserved between the mouse and human genome [13]. A later study [14] showed gene expression to be regulated at a region-wide level in the human genome. Insertion of green fluorescent protein (GFP) reporter constructs at 90 different chromosomal positions in the human genome showed that gene transcription was regulated through a novel region-wide regulatory mechanism as well as via specific transcription factors, thereby demonstrating dual mechanisms in the regulation of gene transcription.

Regional differences in gene expression have been found in two distinct clades (mammals and flies) of the metazoan phylogeny, suggesting a common mechanism of regulation of transcription in all animals. Other characteristics of eukaryotic genomes such as gene density and recombination have also been implied to exhibit domain-like features [15]. In addition, levels of gene expression have been found to correlate with time of chromatin replication during the cell cycle, i.e. the early replication of actively expressed regions of the genome [15]. Striking in this respect is the observed location of gene-dense and highly expressed chromosomes towards the center of the nucleus and the location of gene-poor and weakly expressed chromosomes towards the nuclear envelope in both human [16] and chicken cells [17]. Furthermore, in chicken, this spatial organization seems to correlate with chromosome size [17].

The chicken genome sequence, published in 2004, was the first non-mammalian amniote genome to become available [18]; its karyotype (2*n *= 78) consists of 38 autosomes and one pair of sex chromosomes, with the female being the heterogametic sex (ZW female, ZZ male). Thus far, there are 31 known chromosomes assembled in the chicken genome, including six macro-chromosomes (GGA1-5, Z), five intermediate-chromosomes (GGA6-10) and twenty micro-chromosomes (GGA11-28, 32, W) [18]. The existence of micro-chromosomes is one of the interesting features of the chicken genome [19], micro-chromosomes are also found in some primitive amphibians [20,21] and most reptiles [22]. Besides the huge differences on sizes, microchromosomes also exhibit higher gene density, smaller gene size, and higher recombination rates compared with those in macrochromosomes [18,23]. As the best-studied bird genome currently available, and the closest sequenced genome relative to mammals, the chicken genome is an important resource for comparative genomics, including comparative studies on gene transcription.

To investigate regional effects on transcription in birds, we analyzed chicken gene expression data across a number of different tissues to address three major questions: (i) if there are regional differences in the regulation of transcription in the chicken genome, (ii) if these regions are conserved during evolution, and (iii) the characteristics of these genomic regions in the chicken.

## Results

### Gene expression data

**Figure 1:**
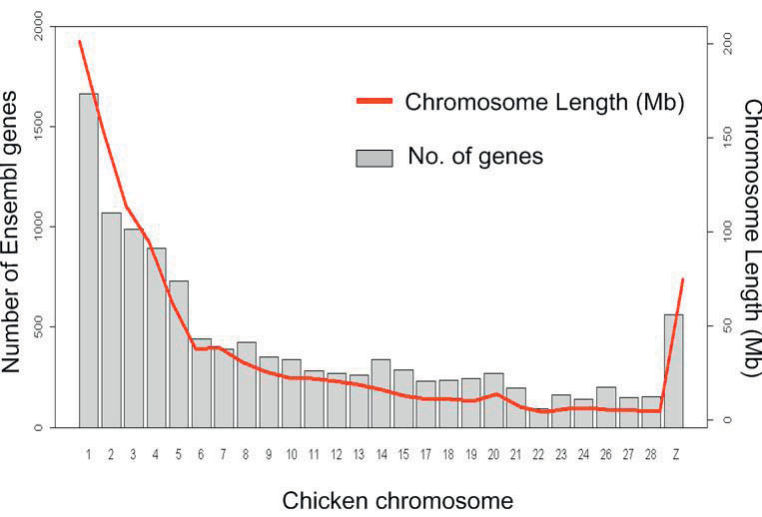
**Distribution of genes on individual chicken chromosomes**. The number of Ensembl genes on each chicken chromosome used in the analysis is shown on the y-axis on the left; the y-axis on the right shows the size of the individual chromosomes.

Eight different chicken tissues were used for the analysis of whole genome gene expression profiles using chicken 20 k oligonucleotide microarrays (GEO [24] accession GPL8861, http://www.ncbi.nlm.nih.gov/geo/query/acc.cgi?token=tjwjpscyceqawjk&acc=GPL8861). All array probes were designed from known transcripts and ESTs based on the chicken genome assembly WASHUC1 (Dec. 2004), and a stringent selection of probes was performed before the analysis. A total of 7,477 probes failed to map to unique chicken Ensembl genes, and these were excluded to avoid the introduction of additional noise into the analysis. In total, 11,361 chicken Ensembl gene IDs located on 27 chromosomes were included in the expression study. These 27 chromosomes cover over 90% of the chicken genome, and include all macro-chromosomes and many of the micro-chromosomes. The number of Ensembl genes on each of these chromosomes is shown in Figure 1. On average, about 70% of all the known ensemble genes on each of these 27 chromosomes were included in this analysis.

In this study, we define the chicken transcriptome map as the median expression levels of the 11,361 chicken Ensembl genes across eight tissues on 27 chromosomes. The start position of the first Ensembl gene and the end position of the last Ensembl gene on each chromosome were considered the start and end of each chicken chromosome. The combined size of the chromosomal sequences analyzed in this study is 1,022,830,111 bp, which covers 97% of the total length of build 2 (WASHUC2, May 2006) of the chicken (*Gallus gallus*) genome.

### Regional differences of transcription in the chicken genome

**Figure 2:**
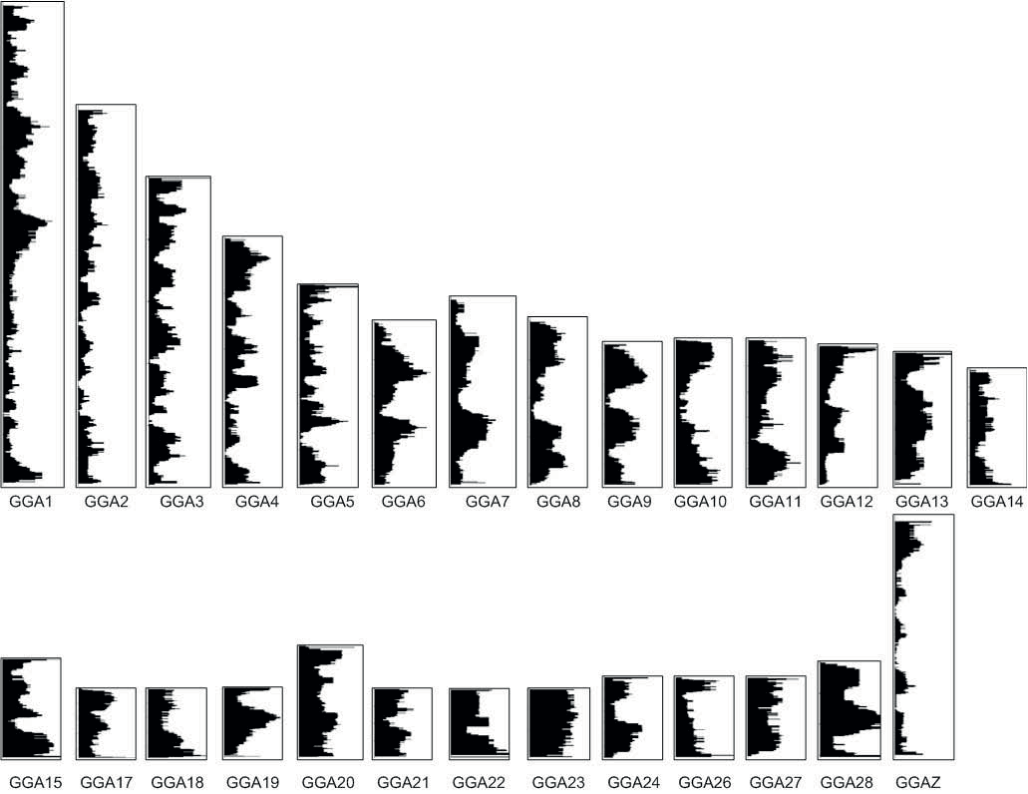
**Regional clusters of highly expressed genes in the chicken genome**. Gene expression is plotted for chicken chromosomes 1-15, 17-24, 26-28, and Z. The expression values are plotted as a moving window with a size of 39 genes to calculate the running median along the chromosomes. The log2 transformed intensities of green channel are shown; the start of the chromosomes correspond with the top of the plot, and the window width indicates the expression levels, ranging between 6.6-8.3 (log2 scale).

**Figure 3:**
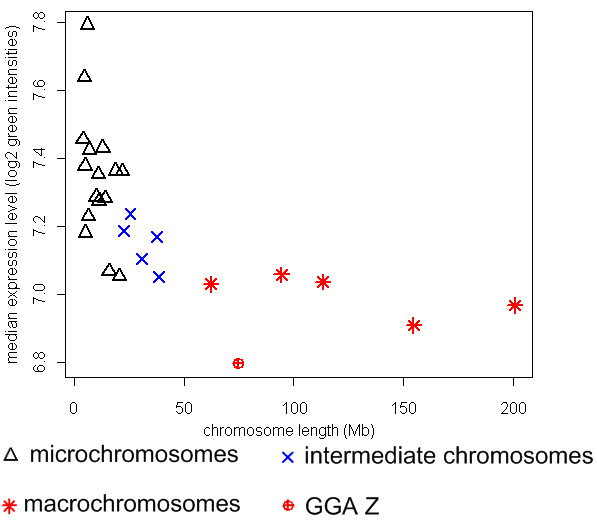
**Relationship between median expression levels and chromosome length (correlation = -0.67, Pearson correlation)**.

To create the chicken transcriptome map, the Ensembl genes were ordered based on the middle positions of the genes on each chromosome, and a robust scatter plot smoothing (running median) technique was applied to the median expression values of the genes on each chromosome (see Materials and Methods for details). The resulting transcriptome map revealed clusters of highly expressed genes on all chicken chromosomes (Figure 2). Marked differences were observed in the overall expression levels of the different chicken chromosomes, with GGA 2, GGA14 and GGAZ showing relatively lower overall gene expression compared to the other chromosomes. Furthermore, the gene expression levels of the micro-chromosomes were observed to be higher than those of intermediate- and macro-chromosomes; the median expression level of each chromosome was observed to decrease with increased chromosome size (Figure 3). Interestingly, the sex chromosome GGAZ shows an extremely low median expression level.

To further investigate the unequal distribution of gene transcription activity along chicken chromosomes, we selected regions with clusters of the most highly expressed genes and regions with clusters of most lowly expressed genes, such that each region type covered approximately ten percent of the chicken genome. To be consistent with previous studies in humans [8,9], here we use the terms "RIDGE" and "anti-RIDGE" to refer to regions showing the highest and lowest expression levels, respectively, in the chicken genome. Similar to Caron et al. [8], we define RIDGEs in the chicken genome as genomic regions with at least 10 consecutive running medians larger than 1.19 times the median expression of the chicken transcriptome, i.e. all 11,361 Ensembl genes. With a running median of a window size of 39 genes, we identified 64 RIDGEs in the chicken genome that cover approximately 10% of the genome. Using the same window size, we identified 27 anti-RIDGEs, which cover approximately 10% of the chicken genome; these anti-RIDGEs are defined as genomic regions with at least 10 consecutive running medians smaller than 0.78 times the median expression of the chicken transcriptome. The total number of Ensembl genes located in RIDGEs and anti-RIDGEs is 3,260 and 1,051, respectively. The mean of the median expression values of genes located in RIDGEs across the tissue panel is approximately 1.8 times higher than that of genes in anti-RIDGEs (Additional file 1). More detailed information of RIDGEs and anti-RIDGEs can be found in Additional file 1.

**Figure 4:**
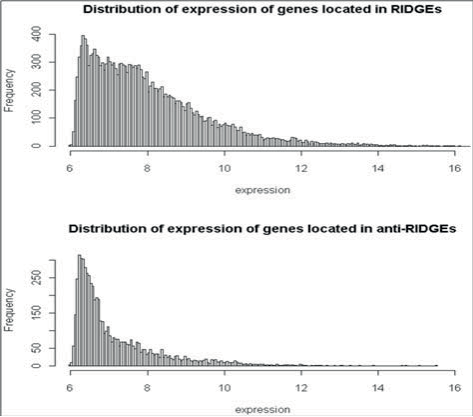
**Histograms of gene expression values across 8 tissues for genes in RIDGEs and anti-RIDGES**. Gene expression on the x-axis is the log2 transformed intensity of the green channel.

The distribution of the expression of the genes located in RIDGEs and anti-RIDGEs is shown in Figure 4. The majority of genes in anti-RIDGEs is below 7 (the log2 transformed intensities of the green channel). This is in strong contrast with the distribution observed for RIDGEs, which show a much broader distribution; furthermore, the majority of genes in RIDGEs show an expression above 7 (the log2 transformed intensities of the green channel).

### Transcriptome maps in different tissues are highly correlated

**Figure 5:**
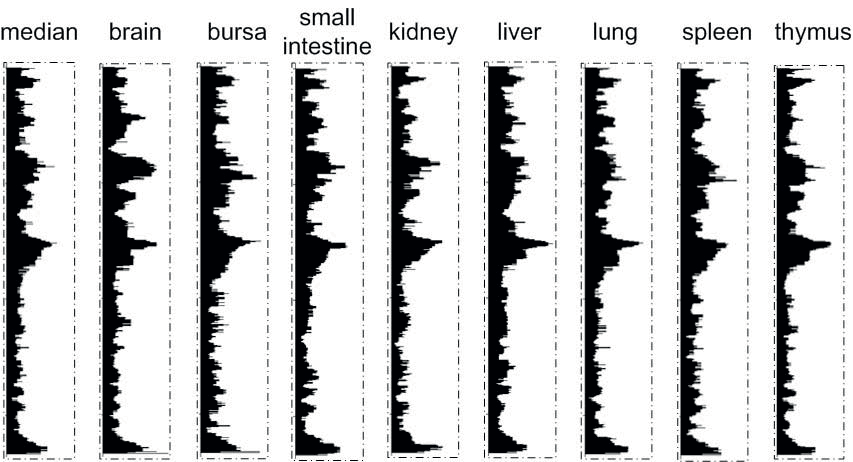
**Transcriptome maps of chromosome 1 for different tissue types, the expression values are plotted as a moving window with a size of 39 genes to calculate the running median along the chicken chromosome 1**. the start of the chromosomes correspond with the top of the plot, and the window width indicates the expression levels, ranging between 6.6-8.3 (log2 scale).

To next evaluate transcriptome maps of different types of tissues, we created transcriptome maps for each individual tissue type by applying a running median on expression values within each tissue using a window size of 39 genes. Chromosome 1 is shown in Figure 5 as an example, and the transcriptome maps for the different tissues were observed to be very similar. We performed a correlation test between the transcriptome map created using the median expression values across the eight tissues and the transcriptome maps created using the expression values from each tissue type. All transcriptome maps are highly correlated, with an average correlation of 0.88. All pair-wise correlations were highly significant, with p-values less than 2.2 × 10^-16^. (All pair-wise correlations between the tissue-specific transcriptome maps are shown in Additional file 2).

### Random permutation tests of RIDGE identification

To test the significance of the number of RIDGEs identified in our analysis, we performed random permutation tests using the same window size and threshold for RIDGE identification. In total, 10,000 random transcriptome maps were generated by permutating the gene orders throughout the genome. The permutation tests, shown in Additional file 3, clearly show that the number of RIDGEs identified in our analysis is higher than would have been expected merely by chance (i.e. that 4.7% of random permutations gave higher numbers of RIDGEs than that observed).

### RIDGEs are relatively conserved between chicken and human

The observation that highly expressed genes tend to be clustered within RIDGEs in the chicken as well as the human genome suggests a conserved functional organization of the genome of these vertebrates. We therefore decided to assess whether genes in RIDGEs remain associated during evolution. Thus, we consider two different forms of functional constraint. The first possibility is that specific genes within a particular RIDGE need to be co-regulated; in this case, one would expect relatively few syntenic breaks to occur within the RIDGEs. The other possibility is that genes do not need to co-localize with specific genes, but rather remain spatially associated with other highly expressed genes in general. In this case, one would expect syntenic breaks to occur specifically between two different RIDGEs. Random rearrangements of RIDGEs and anti-RIDGEs, on the other hand, would reduce the clustering of genes, and therefore abolish the effect of regional regulation of transcription. First we tested if the observed RIDGEs were less prone to be broken down during evolution from chicken to human. Previous studies comparing the human, mouse, rat, and chicken genomes identified a total of 586 conserved synteny blocks [25]. Because the identification of these synteny blocks was based on chicken genome assembly WASHUC1 (Dec. 2004), we mapped the ends of these syntenic blocks to the current chicken genome assembly (WASHUC2, May 2006) (Additional file 4), and considered each end as an evolutionary break point. In total, we mapped 1130 break points on the WASHUC2 chicken genome assembly; we found 253 break points within RIDGEs, and 50 break points within anti-RIDGEs. Chi-square tests showed a significantly higher average number of break points in RIDGEs compared to regions outside RIDGEs (p value < 2.2 × 10^-16^) and a significantly lower number of break points in anti-RIDGEs compared to regions outside anti-RIDGEs (p value = 4.18 × 10^-10^) (Additional file 5).

To compare the transcriptome maps between chicken and human, we downloaded human gene expression data for the same types of tissues (see Materials and Methods) from the Human Transcriptome Map website [26]. Using the median of the expression values across the seven human tissues for each human gene, we performed an identical analysis on the human data as the chicken expression data to identify RIDGEs and anti-RIDGEs in the human genome. Similar to the chicken, in the human genome, RIDGEs and anti-RIDGEs each cover about ten percent of the genome. Defining the syntenic break points in the human genome using data described by Bourque et al. [25], we found a total of 143 and 86 break points in RIDGEs and anti-RIDGEs, respectively. Again, similar to results seen in the chicken, chi-square tests show a higher average number of break points in RIDGEs compared to regions outside of RIDGEs (p value = 0.01) and a lower number of break points in anti-RIDGEs compared to outside anti-RIDGEs (p value = 0.002) (Additional file 5).

We identified 46 RIDGE-to-RIDGE break points and 11 anti-RIDGE-to-anti-RIDGE break points between the chicken and human genomes. Chi-square tests showed a significantly higher number of RIDGE-to-RIDGE break points between the chicken and human genomes (p value < 2.2 × 10^-16^) compared to that expected by chance, and no significant difference in the number of anti-RIDGE-to-anti-RIDGE break points (p value = 0.8).

### Genomic characteristics of RIDGEs and anti-RIDGEs in chicken

**Figure 6:**
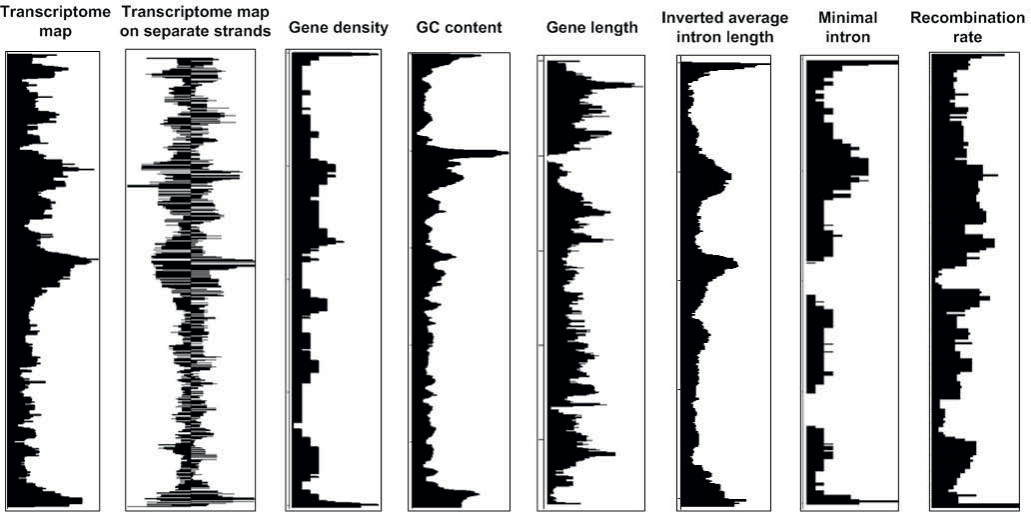
**Whole-chromosome view of (a) transcriptome map **(plotting running medians of gene expression values along chromosome 1 with window size of 39 genes); **(b) transcriptome map on separate strands **(plotting running medians of gene expression values on separate strands with window size of 19 genes on each individual strand (left side: + strand; right side: - strand) along chromosome 1); **(c) gene density **(gene density was defined as number of genes per 100 kb genomic region, running medians of gene densities with window size 39 gene were plotted along chromosome 1); **(d) GC content**; **(e) gene length**; **(f) average intron length **(GC content, gene length, and average intron length were calculated for each gene, the running medians of values for those three features with a window size of 39 genes were plotted along chromosome 1); **(g) "minimal intron" density **(the minimal intron here were defined as introns sizing from 50 to 150 bp, and minimal intron density was defined as the number of minimal introns per 500 kb genomic region, then the running medians of minimal intron intensities with window size of 39 genes were ploted along chromosome 1); and **(h) recombination rate **(recombination rate data of chicken chromosome 1 was obtained from previous study by Groenen et al.[25], and plotted in the same way as described by Groenen et al.) plotted on chicken chromosome one. The start of the chromosome corresponds with the top of the plot.

**Figure 7:**
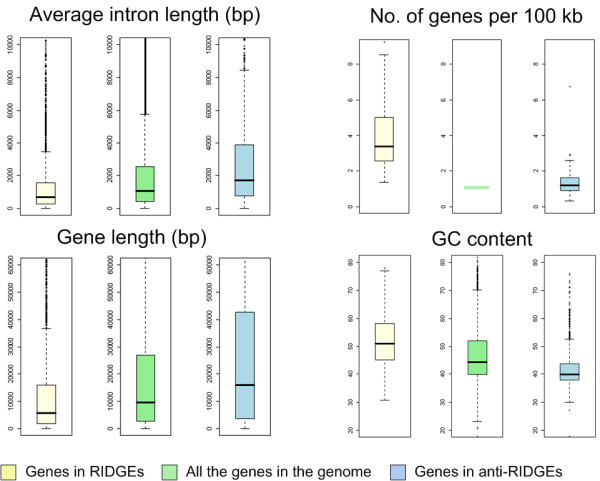
**Boxplot of average intron length, gene length, gene density (number of genes per 100 kb) and GC content for genes in RIDGEs, anti-RIDGEs, and the complete chicken genome**. The middle line of each box represents the median values. The edges of each box represent the first and third quartile values.

Next we evaluated whether RIDGEs and anti-RIDGEs were associated with other genome characteristics. Positive correlations were found between chicken transcriptome map and gene density (p value < 2.2 × 10^-16^), GC content (p value < 2.2 × 10^-16^) and average intron length (p value < 2.2 × 10^-16^). As an example, the whole chromosome views of the transcriptome map, gene density, GC content, gene length, average intron length and recombination rate are shown for chromosome 1 (Figure 6); these various parameters were similar in RIDGEs and anti-RIDGEs. To further investigate the specific genomic characteristics of RIDGEs and anti-RIDGEs, we compared the average intron length (averaged intron length of all transcripts per gene), gene length (genomic length), gene density (number of genes per 100 kb), and GC content between genes located in RIDGEs and anti-RIDGEs (Figure 7). Compared to the entire chicken genome, RIDGEs, on average, harbor genes with shorter average intron length (p value < 2.2 × 10^-16^), shorter gene length (p value < 2.2 × 10^-16^), and a higher GC content (p value < 2.2 × 10^-16^). Anti-RIDGEs, on the other hand, show opposite trends, with genes with longer average intron length (p value < 2.2 × 10^-16^), longer gene length (p value < 2.2 × 10^-16^), and lower GC content (p value < 2.2 × 10^-16^). Furthermore, RIDGEs also have a significantly higher gene density (p value = 1.29 × 10^-9^) than anti-RIDGEs.

### Gene Ontology term enrichment analysis for genes in RIDGEs and anti-RIDGEs

Our results indicate that RIDGEs are relatively conserved between human and chicken. Assuming RIDGEs are the result of evolutionary events favoring the clustering of genes with higher expression levels, one can hypothesize that genes within RIDGEs may share similar functions or biological pathways. To investigate this possibility, we performed Gene Ontology (GO) [27] term enrichment analysis on genes located in RIDGEs and anti-RIDGEs using R package GOstats [28]. However, no significant GOBP terms (the minimum FDR of all three tests is 0.4) were found for genes in RIDGEs and anti-RIDGEs after correcting for multiple testing (Additional file 6).

## Discussion

### Gene expression data

The annotated genes on the array platform used in this study cover most of the current chicken genome assembly. The number of genes analyzed on each chromosome is also in good proportion with chromosome length (Figure 1), which suggests against a bias in the analysis due to uneven distribution of the genes in the chicken genome. We chose to exclude chromosome 16 and 25 from our analysis, as only 24 and 59 Ensembl genes are represented on the array; this number is too low to identify any meaningful high or low expressing regions with the window size of 39 genes used in this analysis.

### No major effect of different tissues on chicken transcriptome map

We observed high correlations (average correlation = 0.88) among the different transcriptome maps based on the expression data from the eight different individual tissues as well as between these transcriptome maps and the transcriptome map of the combined expression data of all eight tissues. This indicates that use of the median expression value or the expression values from individual tissues only has a minor effect on the transcriptome maps and on the identification of RIDGEs and anti-RIDGEs. This shows that regional differences in transcription are a general trend in the chicken genome, even among different tissue types.

### Regional differences of transcription in the genome

This is the first study in birds to construct a transcriptome map and to confirm the existence of regional differences on transcription regulation in the chicken genome. RIDGEs have been discovered in several animal species from phylogenetically distinct groups, suggesting that the existence of RIDGEs may be universal in the animal kingdom [8,10-14].

Gierman et al. [14] showed that RIDGEs contain up to 80 genes and can exert an eightfold difference on the expression levels of integrated genes. They found that gene expression levels are not highly correlated to adjacent genes, but instead more correlated to the entire block of up to 80 genes, demonstrating regional effects on gene transcription. The exact mechanism underlying how gene expression occurs in RIDGEs is still unknown. One hypothesis is that evolution favors highly expressed genes to be physically close to each other, as transcription of one gene would help the chromatin of neighboring genes to "open up" during transcription. This hypothesis is in agreement with our observation of no apparent evolutionary constraint on the co-localization of specific genes, whereas we observed specific localization of specific genes within RIDGEs (see below). Goetze et al. [29] showed that RIDGEs in general are less condensed, more irregularly shaped, and are located more closely to the nuclear center than anti-RIDGEs. Furthermore, the chromatin structures of RIDGEs and anti-RIDGEs are largely independent of tissue-specific variations in gene expression and differentiation state. Their discovery again confirms the hypothesis that the different regional effect of gene transcription in RIDGEs and anti-RIDGEs is, at least in part, explained by the chromatin structure of the two types of genomic regions.

### Genomic Characteristics in RIDGEs and anti-RIDGEs in chicken

Many studies have shown that chicken genome characteristics such as recombination frequency, gene density and GC density correlate with chromosome size [18,23]. Our results show a similar trend with regard to the level of gene expression and density of RIDGEs. In the chicken, the median expression values decrease with increased chromosome length (Figure 3), which can only be partly explained by the higher gene density of the micro-chromosomes. Our permutation analysis clearly shows that the organization of genes in clusters of highly expressed genes is not random and suggests a functional mechanism. This is further strengthened by our observation that the same distribution of RIDGEs is seen when both strands of the same chromosome are analyzed separately (Figure 6). This is additional confirmation of region-like regulation of transcription during gene expression, since the opening of chromatin structures during gene expression will affect both strands by facilitating the access of transcription factors to target genes, thus enhancing gene expression in that region. Furthermore, we also found a correlation between the transcriptome maps and gene density, GC content, gene length, average intron length, "minimal intron" density, and recombination rate in the chicken genome (Figure 6). A correlation between recombination rate and GC content in the chicken genome has been recently reported [23], and these authors therefore link recombination rate with the transcriptome map, as reported in the current study. This can be explained by the more open chromatin structure of the transcriptionally active RIDGEs, which would also facilitate recombination within these regions. Furthermore, "minimal introns" have been reported to be GC-rich and to enhance the rate at which mRNA is exported from the cell nucleus [30] (Yu et al. 2002). These findings link the "minimal introns" distribution via GC content with the transcriptome map in the current study. This can be explained, at least in part, by the need for efficient export of highly expressed mRNA from the nucleus. Many genomic characteristics in eukaryotic genomes, such as RIDGEs, early replication and recombination, appear to be linked. RIDGEs are associated with higher expression, higher gene density, higher GC content, shorter gene introns, shorter genes, higher "minimal intron" density, and higher recombination rate (Figure 6). This is congruent in human studies, in which similar correlations were found [9]. Shorter introns and shorter genes in RIDGEs may indicate the need for increased transcription efficiency. Castillo-Davis et al. [31] showed that introns in highly expressed genes are substantially shorter than those in genes that are expressed at low levels in the human genome, and the authors hypothesized that transcription efficiency is enhanced when intron length is shorter. The clustering of highly expressed genes in RIDGEs therefore would result in clustering of genes with, on average, shorter introns. Although GC content, gene density, gene length, average intron length, "minimal intron" distribution and recombination rate are all correlated with gene transcriptional activity in the chicken genome, the exact causative mechanisms of these relationships are still unknown.

### RIDGEs are relatively conserved between chicken and human

In comparing evolutionary break points between RIDGEs and anti-RIDGEs, we found a higher number of break points within RIDGEs than anti-RIDGEs in both the chicken and the human genome. Similar as for recombination, it is possible that the more open chromatin structure within RIDGEs facilitates an increase in the likelihood of rearrangement events, and thus in an increase in the observed syntenic breaks.

Although RIDGEs clearly show an increase in the number of evolutionary break points, we also showed a significantly higher number of RIDGE-to-RIDGE break points between the chicken and human genomes. Hence, although RIDGEs are more prone to be interrupted by evolutionary break points, there still seems to be an evolutionary constraint that favors recombination between RIDGEs, i.e. the resulting parts of a "broken RIDGEs" from one species were more likely to stay together with a part of another broken RIDGE during genome evolution, thereby keeping specific genes together within RIDGEs. In other words genes within a RIDGE in one species are likely to end up in a RIDGE in another species even when syntenic rearrangements occur. There are in total 11,407 1-to-1 human-chicken homolog genes downloaded via biomaRt [32]. Of these genes, 1,351 are located In RIDGEs and 857 genes are located in anti-RIDGEs in the human genome. 27% of these 1-to-1 human-chicken homolog genes (361 out of 1351 genes) located in human RIDGEs are also located in chicken RIDGEs (p-value smaller than 2.2 × 10^-16^, Chi-square tests). This again supports our hypothesis that genes within a RIDGE in one species are likely to end up in a RIDGE in another species.

This result suggests that the clustering of specific genes is not so much important, but rather the clustering of any genes that are highly expressed. The relative low number of syntenic breaks within anti-RIDGEs, on the other hand, might be linked to another feature of vertebrate chromosomes, namely the occurrence of regions with a relatively low number of genes, so called "gene deserts" [33]. In particular, the so-called "stable gene deserts" colocalize with developmentally active genes and genes coding for transcription factors, both gene types that generally show relatively low levels of expression. These "stable gene deserts" showed extremely low numbers of syntenic breaks [33].

Our results clearly show the existence of a higher level organization of the vertebrate genome affecting not only the expression of genes but also other features such as recombination and genome rearrangements during evolution.

## Conclusion

This is the first study describing a transcriptome map in birds. This study has revealed regional regulation of gene expression in chicken that is consistent with previous studies in flies and mammals [8,10,12]. Since features correlating with high regional transcription are more pronounced in the microchromosomes leading to overall higher expression compared to genes on the macrochromosomes. Our analysis on evolutionary break points shows that the regional regulation of gene transcription is relatively conserved between chicken and human. Given the evolutionary position of chicken on the phylogenetic tree, our results provide a unique perspective for future comparative studies on transcriptome maps between vertebrate species.

## Methods

### Gene expression data

The gene expression data used in this analysis was obtained from a gene expression survey in chicken brain, bursa of Fabricius, kidney, liver, lung, small intestine, spleen and thymus, using the chicken 20 k oligonucleotide microarray (see below). Five biological replicates were used for each tissue type, resulting in a total of 40 arrays. Each individual sample was compared to the pooled reference, and data was normalized using the R [34] package limma [35]. The mean expression value for each Ensembl gene was calculated for each tissue type, and the average expression value of each Ensembl gene was determined by calculating the median expression values across all eight tissues.

The microarray data have been deposited in the Gene Expression Omnibus (GEO) public repository [24]. The accession number for the series is GSE17108, and the sample series can be retrieved with accession numbers from GSM427873 to GSM427912. The sample series contains the raw data (median signal) of each Cy5 (red) and Cy3 (green) channels as well as the normalized data for each microarray.

### Chicken 20 k array platform and oligonucleotide probe re-annotation

The chicken 20 k array was obtained from ARK-Genomics [36]. The array design has been published in Gene Expression Omnibus with the platform name GPL8861 http://www.ncbi.nlm.nih.gov/geo/query/acc.cgi?token=tjwjpscyceqawjk&acc=GPL8861.

The probe sequences of the chicken 20 k oligonucleotide microarray used in this study were designed based on chicken genome assembly WASHUC1 (Dec. 2004), and all sequences were mapped to the chicken genome assembly WASHUC2. An updated array re-annotation file based on Ensembl 50 is available at EADGENE Oligo Set Annotation Files homepage [37]. Of the total 20,460 oligonucleotide probes on the chicken 20 k array, 13,431 mapped to unique locations in the chicken genome. All the probes for genes that mapped to chromosome "unknown" were excluded in the analysis, and all probes for genes on chromosome 16, 25, and W were excluded due to the very low number of probes that mapped to those chromosomes. For probes that mapped to the same known Ensembl gene ID [38], the expression data were averaged and assigned to the Ensembl gene. In total, in this study, 12,983 oligo probes were used that mapped to 11,361 unique chicken Ensembl gene IDs located on 27 chromosomes.

### Identification of RIDGEs in the chicken genome

Individual gene expression data was ordered according to the middle position of the gene. A Robust Scatter Plot Smoothing (function *runmed *in R package stats) technique was applied to each chromosome separately, with a window size of 39 genes, i.e. the expression value of each gene was replaced by the median expression value of the neighboring 39 genes. Similar to the definition for RIDGEs in humans [8], here we defined a RIDGE by window size for calculating median expression, minimum length of the run, and the threshold for the lower limit of the median. The selection of window size of 39 genes was based on the following two points: 1) Permutation analysis performed by both Caron et al. [8] and our analysis indicated a window size of 39 genes gives a reasonable number of RIDGEs; 2) To be able to compare the results of RIDGE identification between human and chicken, we decided to use the same threshold as described by Caron et al. The bigger the window size is, the smaller number of RIDGEs will be identified as indicated in the permutation results in Additional file 3.

The threshold for RIDGEs was set to 1.19 times the genomic median value (the data are log2 transformed, and the values used here is the running median values of a window size of 39 genes) along the length of a run of at least 10 median values. The threshold used for anti-RIDGEs was a median expression of 0.78 times the genomic median. The thresholds used for the classification of the RIDGEs and anti-RIDGEs were chosen such that RIDGEs and anti-RIDGEs each cover 10% of the genome.

### Correlation analysis between tissue-specific transcriptome maps

Spearman rank correlation test was performed to test for pairwise correlations among the transcriptome maps on all the chromosomes (applied to the running median with window size of 39 genes). The running median expression values are not normally distributed, and the non-parametric Spearman correlation test was used on the ranks of the paired transcriptome maps.

### Random permutation tests for RIDGE identification in chicken

Random permutation tests were done in R by permuting the genomic locations of Ensembl genes and repeating the RIDGE analysis 10,000 times to create 10,000 random transcriptome maps. The number of RIDGEs identified in these 10,000 random transcriptome maps was compared to the actual number of identified RIDGEs in this analysis using the same threshold.

### Syntenic break points

Human-chicken synteny block data from Bourque et al. [25] was used in this study, and genomic locations of synteny blocks from assembly WASHUC1 (Dec 2004) were mapped to assembly WASHUC2 (May 2006) using BLAT (see Additional file 4). Each end of every syntenic block was considered a break point, and the number of break points in RIDGEs and anti-RIDGEs was subsequently summarized.

### Human gene expression data

Human Transcriptome Map data was downloaded from the HTM website [26]. We selected Affymetrix U133A human whole genome array data from seven tissues (thymus, spleen, lung, small intestine, brain, liver, and kidney) from a healthy individual; data (normalized data) was log2 transformed and the median expression value across the seven different tissues was used to build the transcriptome map. RIDGEs and anti-RIDGEs were identified using the same approach as for the chicken data.

### Genome characteristics of RIDGEs and anti-RIDGEs in chicken

Genomic location, transcript length, exon number and GC content for the individual Ensembl chicken genes were downloaded from the Ensembl genome database using biomaRt [32]. The averaged intron length was calculated by averaging the intron length of all transcripts per gene. The statistical test for differences in average intron length, gene length, gene density, and GC content between RIDGEs and anti-RIDGEs was performed using Wilcoxon rank-sum test (function Wilcox.test function in R package stats).

### GO term enrichment analysis

GO term enrichment analysis was performed using R package Gostats [28]. The conditional algorithm was used for the hypergeometric test. The gene annotation package for the GOstats analysis was built using R package AnnotationDbi [39]. Mapping of chicken Ensembl gene IDs and other genomic information (e.g. entrezgene) was performed using the R package biomaRt [32].

## List of abbreviations used

MHC: Major Histocompatibility Complex; TF: Transcription Factor; SAGE: Serial Analysis of Gene Expression; RIDGE: Regions of Increased Gene Expression; GFP: Green Fluorescent Protein; EST: expressed sequence tag; GO: Gene Ontology.

## Authors' contributions

HN carried out the experiment, performed data analysis and drafted the manuscript, JB helped with statistical analysis of this work, HJM, RC, and MG helped with interpretation of the results, all authors were involved in improving the manuscript. The final version of the manuscript was approved by all the authors.

## Supplementary Material

Additional file 1 **Genomic location of RIDGEs and anti-RIDGEs**. Genomic location of RIDGEs and anti-RIDGEs identified in the chicken genome in this study. Click here for file

Additional file 2 **Correlations of transcriptome maps in different tissues**. All pairwise correlations between the tissue-specific transcriptome maps. Click here for file

Additional file 3 **Random permutation test**. Random permutation test results for RIDGE identification with different window sizes. Click here for file

Additional file 4 **Positions of the synteny block in the chicken genome**. Genomic positions of the ends of the synteny block on genome build WASHUC2. Click here for file

Additional file 5 **Evolutionary breaks within RIDGEs and anti-RIDGEs**. Chi-square test of evolutionary break points within RIDGEs and anti-RIDGEs. Click here for file

Additional file 6 **GO enrichment analysis for genes in RIDGEs and anti-RIDGEs**. Enriched GOBP terms for all genes located within RIDGEs and anti-RIDGEs. BY: adjusted *p*-values for the Benjamini & Yekutieli step-up FDR controlling procedure. Click here for file

## References

[B1] Kielman MF Smits R Devi TS Fodde R Bernini LF Homology of a 130-kb region enclosing the alpha-globin gene cluster, the alpha-locus controlling region, and two non-globin genes in human and mouse Mamm Genome 1993 4 6 314 323 10.1007/BF00357090 8318735

[B2] The MHC sequencing consortium Complete sequence and gene map of a human major histocompatibility complex Nature 1999 401 6756 921 923 10.1038/44853 10553908

[B3] Amores A Force A Yan YL Joly L Amemiya C Fritz A Ho RK Langeland J Prince V Wang YL Westerfield M Ekker M Postlethwait JH Zebrafish hox clusters and vertebrate genome evolution Science 1998 282 5394 1711 1714 10.1126/science.282.5394.1711 9831563

[B4] Garcia-Fernandez J The genesis and evolution of homeobox gene clusters Nat Rev Genet 2005 6 12 881 892 10.1038/nrg1723 16341069

[B5] Hurst LD Pal C Lercher MJ The evolutionary dynamics of eukaryotic gene order Nat Rev Genet 2004 5 4 299 310 10.1038/nrg1319 15131653

[B6] Sproul D Gilbert N Bickmore WA The role of chromatin structure in regulating the expression of clustered genes Nat Rev Genet 2005 6 10 775 781 10.1038/nrg1688 16160692

[B7] Michalak P Coexpression, coregulation, and cofunctionality of neighboring genes in eukaryotic genomes Genomics 2008 91 3 243 248 10.1016/j.ygeno.2007.11.002 18082363

[B8] Caron H van Schaik B Mee M van der Baas F Riggins G van Sluis P Hermus MC van Asperen R Boon K Voute PA Heisterkamp S van Kampen A Versteeg R The human transcriptome map: clustering of highly expressed genes in chromosomal domains Science 2001 291 5507 1289 1292 10.1126/science.1056794 11181992

[B9] Versteeg R van Schaik BD van Batenburg MF Roos M Monajemi R Caron H Bussemaker HJ van Kampen AH The human transcriptome map reveals extremes in gene density, intron length, GC content, and repeat pattern for domains of highly and weakly expressed genes Genome Res 2003 13 9 1998 2004 10.1101/gr.1649303 12915492 PMC403669

[B10] Spellman PT Rubin GM Evidence for large domains of similarly expressed genes in the Drosophila genome J Biol 2002 1 1 5 10.1186/1475-4924-1-5 12144710 PMC117248

[B11] Boutanaev AM Kalmykova AI Shevelyov YY Nurminsky DI Large clusters of co-expressed genes in the Drosophila genome Nature 2002 420 6916 666 669 10.1038/nature01216 12478293

[B12] Mijalski T Harder A Halder T Kersten M Horsch M Strom TM Liebscher HV Lottspeich F de Angelis MH Beckers J Identification of coexpressed gene clusters in a comparative analysis of transcriptome and proteome in mouse tissues Proc Natl Acad Sci USA 2005 102 24 8621 8626 10.1073/pnas.0407672102 15939889 PMC1143582

[B13] Singer GA Lloyd AT Huminiecki LB Wolfe KH Clusters of co-expressed genes in mammalian genomes are conserved by natural selection Mol Biol Evol 2005 22 3 767 775 10.1093/molbev/msi062 15574806

[B14] Gierman HJ Indemans MH Koster J Goetze S Seppen J Geerts D van Driel R Versteeg R Domain-wide regulation of gene expression in the human genome Genome Res 2007 17 9 1286 1295 10.1101/gr.6276007 17693573 PMC1950897

[B15] Chakalova L Debrand E Mitchell JA Osborne CS Fraser P Replication and transcription: shaping the landscape of the genome Nat Rev Genet 2005 6 9 669 677 10.1038/nrg1673 16094312

[B16] Croft JA Bridger JM Boyle S Perry P Teague P Bickmore WA Differences in the localization and morphology of chromosomes in the human nucleus J Cell Biol 1999 145 6 1119 1131 10.1083/jcb.145.6.1119 10366586 PMC2133153

[B17] Habermann FA Cremer M Walter J Kreth G von Hase J Bauer K Wienberg J Cremer C Cremer T Solovei I Arrangements of macro- and microchromosomes in chicken cells Chromosome Res 2001 9 7 569 584 10.1023/A:1012447318535 11721954

[B18] International Chicken Genome Sequencing Consortium Sequence and comparative analysis of the chicken genome provide unique perspectives on vertebrate evolution Nature 2004 432 7018 695 716 10.1038/nature03154 15592404

[B19] Rodionov AV Micro vs. macro: structural-functional organization of avian micro- and macrochromosomes Genetika 1996 32 5 597 608 8755033

[B20] Morescalchi A Odierna G Olmo E Karyological relationships between the Cyptobranchid salamanders Specialia 1977 15 1579

[B21] Morescalchi A Odierna G Olmo E Karyology of the primitive salamanders, family Hynobiidae Experientia 1979 35 1434 1436 10.1007/BF01962768 510468

[B22] Mengden GA Stock AD Chromosomal evolution in Serpentes: a comparison of G and C chromosome banding patterns of some Colubrid and Boid genera Chromosoma 1980 79 53 64 10.1007/BF00328472

[B23] Groenen MA Wahlberg P Foglio M Cheng HH Megens HJ Crooijmans RP Besnier F Lathrop M Muir WM Wong GK Gut I Andersson L A high-density SNP-based linkage map of the chicken genome reveals sequence features correlated with recombination rate Genome Res 2009 19 3 510 519 10.1101/gr.086538.108 19088305 PMC2661806

[B24] National Center for Biotechnology Information (NCBI) Gene Expression Omnibus http://www.ncbi.nlm.nih.gov/geo/

[B25] Bourque G Zdobnov EM Bork P Pevzner PA Tesler G Comparative architectures of mammalian and chicken genomes reveal highly variable rates of genomic rearrangements across different lineages Genome Res 2005 15 1 98 110 10.1101/gr.3002305 15590940 PMC540283

[B26] Human Transcriptome Map website http://bioinfo.amc.uva.nl/HTMseq/

[B27] Gene Ontology website http://www.geneontology.org/

[B28] Falcon S Gentleman R Using GOstats to test gene lists for GO term association Bioinformatics 2007 23 2 257 258 10.1093/bioinformatics/btl567 17098774

[B29] Goetze S Mateos-Langerak J Gierman HJ de Leeuw W Giromus O Indemans MH Koster J Ondrej V Versteeg R van Driel R The three-dimensional structure of human interphase chromosomes is related to the transcriptome map Mol Cell Biol 2007 27 12 4475 4487 10.1128/MCB.00208-07 17420274 PMC1900058

[B30] Yu J Yang Z Kibukawa M Paddock M Passey DA Wong GK Minimal introns are not "junk" Genome Res 2002 12 8 1185 1189 10.1101/gr.224602 12176926 PMC186636

[B31] Castillo-Davis CI Mekhedov SL Hartl DL Koonin EV Kondrashov FA Selection for short introns in highly expressed genes Nat Genet 2002 31 4 415 418 12134150 10.1038/ng940

[B32] Durinck S Moreau Y Kasprzyk A Davis S De Moor B Brazma A Huber W BioMart and Bioconductor: a powerful link between biological databases and microarray data analysis Bioinformatics 2005 21 16 3439 3440 10.1093/bioinformatics/bti525 16082012

[B33] Ovcharenko I Loots GG Nobrega MA Hardison RC Miller W Stubbs L Evolution and functional classification of vertebrate gene deserts Genome Res 2005 15 1 137 145 10.1101/gr.3015505 15590943 PMC540279

[B34] R Development Core Team R: A language and environment for statistical computing 2008 R Foundation for Statistical Computing, Vienna, Austria

[B35] Smyth GK Gentleman R, Carey V, Dudoit S, Irizarry R, Huber W Limma: linear models for microarray data Bioinformatics and Computational Biology Solutions using R and Bioconductor 2005 New York: Springer 397 420 full_text

[B36] Ark-Genomics, Roslin institute, UK http://www.ark-genomics.org/

[B37] EADGENE Oligo Set Annotation Files homepage http://www.eadgene.info/TheProject/Integration/BiologicalresourcesandfacilitiesWP1/EADGENEOligoSetsAnnotationFiles/tabid/324/Default.aspx

[B38] Ensembl Genome Database http://www.ensembl.org/

[B39] Pages H Carlson M Falcon S Li N AnnotationDbi: Annotation Database Interface R package version 1.4.0 2008

